# Interactions “*Candidatus* Liberibacter solanacearum”—*Bactericera cockerelli*: Haplotype Effect on Vector Fitness and Gene Expression Analyses

**DOI:** 10.3389/fcimb.2016.00062

**Published:** 2016-06-09

**Authors:** Jianxiu Yao, Panatda Saenkham, Julien Levy, Freddy Ibanez, Christophe Noroy, Azucena Mendoza, Ordom Huot, Damien F. Meyer, Cecilia Tamborindeguy

**Affiliations:** ^1^Department of Entomology, Texas A&M UniversityCollege Station, TX, USA; ^2^Department of Horticultural Sciences, Texas A&M UniversityCollege Station, TX, USA; ^3^CIRAD, UMR CMAEEPetit-Bourg, Guadeloupe, France; ^4^Institut National de la Recherche Agronomique, UMR1309 CMAEEMontpellier, France; ^5^Université des Antilles, Pointe-à-PitreGuadeloupe, France

**Keywords:** psyllid, *Bactericera cockerelli*, *Candidatus* Liberibacter solanacearum, Lso haplotype, effectors

## Abstract

“*Candidatus* Liberibacter solanacearum” (Lso) has emerged as a serious threat world-wide. Five Lso haplotypes have been identified so far. Haplotypes A and B are present in the Americas and/or New Zealand, where they are vectored to solanaceous plants by the potato psyllid, *Bactericera cockerelli* (Šulc) (Hemiptera: Triozidae). The fastidious nature of these pathogens has hindered the study of the interactions with their eukaryotic hosts (vector and plant). To understand the strategies used by these pathogens to infect their vector, the effects of each Lso haplotype (A or B) on psyllid fitness was investigated, and genome-wide transcriptomic and RT-qPCR analyses were performed to evaluate Lso gene expression in association with its vector. Results showed that psyllids infected with haplotype B had significantly lower percentage of nymphal survival compared to psyllids infected with haplotype A. Although overall gene expression across Lso genome was similar between the two Lso haplotypes, differences in the expression of key candidate genes were found. Among the 16 putative type IV effector genes tested, four of them were differentially expressed between Lso haplotypes, while no differences in gene expression were measured by qPCR or transcriptomic analysis for the rest of the genes. This study provides new information regarding the pathogenesis of Lso haplotypes in their insect vector.

## Introduction

“*Candidatus* Liberibacter solanacearum” (Lso) is emerging as a serious pathogen of crops worldwide. Presently, five Lso haplotypes (Lso A, Lso B, Lso C, Lso D, and Lso E) have been identified infecting different crops (Munyaneza et al., 2010; Alfaro-Fernández et al., 2012; Lin et al., 2012; Teresani et al., 2014). The Lso haplotypes were identified using three approaches: single nucleotide polymorphism (SNP) genotyping of the *16S* rRNA, *16S/23S* ISR, and *50S rplJ* and *rplL* ribosomal protein genes (Nelson et al., 2011); multilocus sequence typing markers (MLST; Glynn et al., 2012); and simple sequence repeat (SSR; Lin et al., 2012). Lso A and Lso B are vectored by potato psyllids, *Bactericera cockerelli* (Hemiptera: Triozidae), and are associated with potato Zebra Chip and other solanaceous diseases in the Americas and New Zealand (Munyaneza et al., 2007; Liefting et al., 2008, 2009a,b). Lso C and Lso D are vectored by carrot psyllids, *Trioza apicalis* (Munyaneza et al., 2010) and *Bactericera trigonica* (Alfaro-Fernández et al., 2012), respectively, and are found infecting carrots in Europe. Haplotype E has been identified recently in Spain infecting celery and carrots (Teresani et al., 2014).

Previously, we have shown that Lso negatively affected potato psyllid's fitness. Potato psyllids harboring Lso showed lower oviposition and nymphal survivorship than psyllids harboring no Lso (Nachappa et al., 2012b, 2014).

Lso must exploit its host's cell machinery and avoid host's immune defenses (Vyas et al., 2015). As other vector-borne bacterial pathogens, Lso has to adapt itself to two different environments, the vector and the plant hosts. One way microbial pathogens can hijack different biological processes of the eukaryotic host and create a suitable environment for their survival is by using protein effectors (Sugio et al., 2011; MacLean et al., 2014). For instance, “*Candidatus* Liberibacter asiaticus” secretes a prophage encoded peroxidase that detoxifies H_2_O_2_, and thus potentially suppresses the transcriptional activation of *Rboh* (Jain et al., 2015), which is involved in systemic acquired resistance and innate plant immunity. Although manipulation of plant hosts by *Liberibacter* has been studied, the manipulation of vector at the transcriptomic level has not. Furthermore, how psyllids defend against *Liberibacter* bacteria remains unknown (Nachappa et al., 2012a; Fisher et al., 2014; Reese et al., 2014), but bacterial effectors might play a central role in the psyllid-bacterial interaction.

In this study, effects of Lso A and Lso B on potato psyllid fitness were assessed and Lso genes potentially associated with these effects were identified. These data will pave the way to understand the differences between Lso A and Lso B pathogenicity, and provide insights into the interactions between psyllids and *Liberibacter*.

## Materials and methods

### Plants

All experiments were performed using tomato plants, *Solanum lycopersicum* L. cultivar Moneymaker (Thompson and Morgan Inc., Jackson, NJ). Plants were grown from seed in 2 × 2 inches pots with Sun Gro® Sunshine LP5 mix (Bellevue, WA) and fertilized twice a week with the label rate of Miracle-Gro® Water Soluble Tomato Plant Food (18-18-21 NPK) (Scotts Company, OH). Four-week-old seedlings were transferred to 4 × 4 inches pots, individually. All experiments were performed 1 week after transplant, or when plants had four fully expanded leaves.

### Insects

A Lso-free potato psyllids colony, Northwestern haplotype, was obtained from Dr. Henne, AgriLife Research at Weslaco, in 2013, and reared on tomato plants in insect-proof cages (24 × 13.5 × 13.5 inches, Bioquip, Compton, CA) at room temperature and a photoperiod of 16-h light:8-h dark.

To obtain potato psyllid colonies carrying each of the Lso haplotypes, 6-week-old tomato plants were infected as previously described (Nachappa et al., 2014) using three third instar nymphs from potato psyllid laboratory colonies harboring Lso A and Lso B haplotypes. After a week, nymphs were removed. Three weeks after Lso inoculation, the plants were tested for Lso infection using LsoF/OI2 primers (Li et al., 2009) and for Lso haplotype using Lso SSR-1 primers (Lin et al., 2012). Once plants tested positive for only one of the Lso haplotypes, 50 Lso-free potato psyllid nymphs were transferred onto these plants for a 24-h Lso acquisition access period (AAP). After AAP, nymphs were transferred to healthy plants and maintained for at least two generations (about 2 months) for infection stabilization. These colonies are referred to as LsoA and LsoB colonies. For verification, diagnostic PCRs were performed to detect the presence of Lso and Lso haplotypes regularly.

### Female oviposition

Fifth instar nymphs were collected from each colony (Lso-free, LsoA, and LsoB) and transferred onto different tomato plants. The ensuing newly emerged adults (24 h after emergence) were collected and paired (one female and one male). One pair of insects from each colony was placed on a tomato leaf covered with a mesh cage (9 × 12 cm) for 2 weeks under photoperiod 16 L:8 D at room temperature to allow mating and egg laying. After 2 weeks, all adults were removed; all nymphs and eggs on that leaf were counted and recorded as “two-week female oviposition.” If one of the adults (male or female) died before the 2-week period, the data were excluded from the analysis. This experiment was performed three times. At least 10 couples from each colony were used in this analysis. Presence of Lso and Lso haplotype in each female were further determined after the two-week oviposition period as described before.

### Egg viability and nymphal survivorship

For each colony, life history and life table parameters were determined starting from newly oviposited eggs until all individuals completed development. In this experiment, in order to synchronize insect development, 30 mature adults (15 males and 15 females) from each colony were placed in a mesh cage (9 × 12 cm) on the fourth leaf of 5-week old tomato plants, respectively. After a 24 h-oviposition period, the adults were removed and the number of eggs was recorded. Each day, the number of eggs, nymphs and/or adults were recorded and every newly emerged adult was removed. Egg viability was determined as the percentage of eggs that hatched. The time period between oviposition and first egg hatch was considered egg incubation time. The time period between the first egg hatch and the first adult emergence represented immature development time. Percentage of nymphal survival was determined as the percentage of first instar nymphs that reached adulthood. The experiment had three independent replicates per treatment and was performed twice at room temperature with a photoperiod of 16:8 h (L:D). At the end of each experiment, three females from each replicate were pooled to test for Lso infection and Lso haplotype as described before.

### Transcriptome analyses

Thirty mature adults (15 males and 15 females) from LsoA or LsoB colonies were placed in a mesh cage (9 × 12 cm) on the fourth leaf of 5-week old tomato plants. There were three independent replicates per colony. The insects were allowed a 24 h oviposition period; then the adults were removed from the plants. Progeny was allowed to develop and once young adults emerged, teneral, < 1-day old adults from the LsoA and LsoB colonies were collected daily. Insects were sexed under the dissecting microscope and 30 individuals of each sex were pooled together, flash frozen in liquid nitrogen and stored at −80°C. There was one pool per Lso haplotype, per sex, and per replicate.

RNA samples for Illumina sequencing were extracted and processed as previously published (Ibanez et al., 2014). Briefly, total RNA from each pool of insects was purified using RNeasy Mini kit (Qiagen, Valencia, CA) followed by DNase treatment with RNase-Free DNase (Qiagen, Valencia, CA). Two micrograms of RNA from each sample were pooled into superpools for a total of 6 μg of RNA per sex and per Lso haplotype. Superpooled RNA samples were subjected to ribosomal RNA depletion using RiboMinus Transcriptome Isolation kit (Life Technologies, Carlsbad, CA) combined with 100 pmol of each psyllid specific probes and 100 pmol of bacterial rRNA specific probes from the kit. PolyA RNA was further purified using Dynabeads mRNA Purification kit (Life Technologies, Carlsbad, CA).

PolyA purified RNA and the rest of the RNA (rRNA and polyA depleted, later called depleted RNA) from each superpool were submitted to the Texas AgriLife Genomics and Bioinformatics Core Facility in Texas A&M University for sequencing. Eight libraries (depleted RNA and polyA purified RNA from LsoA males, LsoA females, LsoB males, and LsoB females) were prepared using the TruSeq Library kit (Illumina, San Diego, CA). Both libraries from each sample were combined in a 60:40 ratio (depleted RNA: polyA purified RNA). All samples were sequenced using two lanes of 125 base reads chemistry on Illumina Hi-SEQ system. Sequencing data have been deposited in NCBI GEO's database (GSE81209).

Illumina sequences were processed using the Illumina pipeline. Programs for sequence processing were used to produce the fastq files, sort libraries, and remove the barcodes and adaptors. The Brazos cluster and CyVerse were used for sequence manipulation and CLC genomic workbench 6.04 platform was used for read mapping to Lso haplotype B genome (Lin et al., 2011) and RNA-seq analysis as previously published (Ibanez et al., 2014). For comparison, reads were also mapped to Lso A genome (Thompson et al., 2015). Genes were ranked by RPKM value. Reciprocal blast searches were conducted to evaluate if specific genes were present in Lso A and Lso B genomes. Relative expression of candidate genes was calculated relative to *recA* expression using the formula: RPKM target gene/RPKM recA.

To validate bioinformatics analyses, cDNA synthesis reactions were performed from each total RNA pool (before depletion and superpooling) that were used for Illumina sequencing. Five hundred nanogram of total RNA was reverse-transcribed into cDNA using random hexamers primers and the Verso cDNA Synthesis kit (Thermo Fisher Scientific, Waltham, MA) following the manufacturer's protocol. The RT-qPCR reactions were performed using SensiFAST SYBR Hi-ROX Kit (Bioline, Taunton, MA) according to manufacturer's instructions. Each reaction contained 5 ng of cDNA, 250 nM of each primer (Table 1) and 1X of SYBR Green Master Mix, the volume was adjusted with nuclease-free water to 10 μL. The real-time PCR program was 95°C for 2 min followed by 40 cycles at 95°C for 5 s and 60°C for 30 s. Gene expression was tested in an ABI 7300 real-time PCR thermocycler (Applied Biosystems) using two technical replicates for each synthetized cDNA, with negative controls in each run. The threshold cycles (Ct) values and the efficiency of each primer set for RT-qPCR were determined using LinRegPCR software (Ramakers et al., 2003). The relative expression of each gene [2^−(CTtarget gene−CTnormalizer gene)^] was estimated by normalizing transcript levels of target genes to the internal control gene (Lso *recA*) expression values [Lso *recA* primer as in Ibanez et al., 2014]. For RT-qPCR, four putative effector genes (CKC_RS04230, CKC_RS03915, CKC_RS03550, and CKC_RS04080) were chosen based on transcriptome analyses in which two of them were differentially expressed between LsoA and LsoB, while the other two were not (primers shown in Table 1). Gene expression data from bioinformatics analyses and RT-qPCRs were compared using Pearson's correlation coefficient. Relative expression for each target gene using *recA* as normalizer was calculated using the formulae: RPKM target gene/RPKM recA and 2^−(CTtarget gene−CTnormalizer gene)^.

**Table 1:** **Primers used to validate gene expression of putative effectors**.

**Primer name**	**Sequence (5′–>3′)**	**Amplicon size (bp)**	**PCR efficiency (%)**
**S4TE EFFECTORS**			
CKC_RS05675 F	TGTCTCATTCCGTTGCTTCC	104	93.2
CKC_RS05675 R	CCAATGCCACACTCCGTAATA		
CKC_RS04080 F^*^	GTTACGCCTTGTAGATCCAGAG	104	95.2
CKC_RS04080 R^*^	CTCGCTCTATTTCCTCCGTTATT		
CKC_RS03550 F^*^	CTTTTGCACGCATTAGCAG	144	98.7
CKC_RS03550 R^*^	AACTTCTTCCGGAACACTC		
CKC_RS05565 F	AATGCTGTTTCTGGGGTTG	130	90.2
CKC_RS05565 R	CTAGAGTTAGAACATGCGG		
CKC_RS01780 F	TGGACGTGGTGTTTCCTATTT	131	88.3
CKC_RS01780 R	CCTGCATTTCATGCGCTAATC		
CKC_RS04955 F	AACGGTTCCTCAGGTGGTT	133	93.4
CKC_RS04955 R	GCTTCAGCTGTTGTAGCTT		
CKC_RS03370 F	ATGGCTTCTAGGCGTGTTT	101	93.1
CKC_RS03370 R	CGCCCTCCTCTAACTTGTAATC		
CKC_RS01290 F	AGAACCTGCTCCAGGAATAAAG	92	91.3
CKC_RS01290 R	CTGCAACACGTGCTGAAATAG		
CKC_RS05235 F	GATCGTCCAAACACATGGATAAAC	91	87.2
CKC_RS05235 R	TCCATGCTTCTATGCTGTGAG		
CKC_RS03655 F	CTCGTCGTCTTGCTGCTATT	102	92.7
CKC_RS03655 R	GCAGATGTGCTTTCATAAGTTCC		
CKC_RS02175 F	TCGGCTATATCCAGCCAAATATC	109	89
CKC_RS02175 R	GATCACCATTGATCTTCCGTAGT		
CKC_RS01950 F	GTCGCGCGGGAAGTAATAAA	96	93.6
CKC_RS01950 R	CATGTTCGGCTTCTCGGAAATA		
CKC_RS03915 F^*^	CAATTCCATCAACGCAAG	90	92.8
CKC_RS03915 R^*^	TTTCCGCTGGAGTAGCTT		
CKC_RS00880 F	CACAGCATCTTCCTGAGTTAGT	96	93.7
CKC_RS00880 R	TCTAATCCTCCACGGTGAAATG		
CKC_RS00225 F	TGATGCGCGTGGTATTAGAG	103	94.5
CKC_RS00225 R	GGCCAAGAAGGGAAGGAATA		
CKC_RS00885 F	GGAGGTTGAAGCTCTTAAG	141	91.7
CKC_RS00885 R	TTGGAATCAAACTCTGCCC		
**EFFECTOR WEBSITE**			
CKC_RS04230 F^*^	GTCTAAGTTGCGAATTGCC	149	100
CKC_RS04230 R^*^	AAAAGTCTCGGGTTCATCC		
CKC_RS02605 F	TCATGGGTCGTGCTATGAT	159	92.7
CKC_RS02605 R	AGCCAAAGACATGCTCTTC		

* *primers used to validate bioinformatic analysis of transcriptomic data*.

### Effector prediction and expression

Putative Lso type IV effectors were predicted using the S4TE software (Meyer et al., 2013) using Lso B genome (Lin et al., 2011). Other effectors were identified from the EffectiveDB database (Jehl et al., 2011).

To assess the expression of Lso effector genes in females and males from the LsoA and LsoB colonies, three new pools of thirty 1-day old Lso A and Lso B infected male and female psyllids were collected, respectively. The total RNA and DNA were extracted from each pool by using ZR-Duet DNA/RNA MiniPrep kit (Zymo Research, Irvine, CA) following the protocol from the manufacturer.

Primers were designed using Primer3 (Untergasser et al., 2012; Table 1) using Lso B genome (Lin et al., 2011), before the release of Lso A genome (Thompson et al., 2015). Since at the time the Lso A genome had not been released, primers were validated by qPCR using LsoA and LsoB colony DNA as previously described using 5 ng of each DNA as template.

For effector expression analysis, 500 ng of total RNA from each pool was reverse-transcribed into cDNA using random hexamers primers by Verso cDNA Synthesis kit (Thermo Fisher Scientific, Waltham, MA) following the manufacturer's protocol. The RT-qPCR reactions were performed using SensiFAST SYBR Hi-ROX Kit (Bioline, Taunton, MA) according to manufacturer's instructions. Reactions were performed as described before using three technical replicates for each synthetized cDNA, with negative controls in each run. The threshold cycles (Ct) values and the efficiency of each primer set for RT-qPCR were determined using LinRegPCR software (Ramakers et al., 2003). The relative expression of each gene was estimated by normalizing transcript levels of genes of interest to the internal control gene (Lso *recA*) expression values [2^−(CTtarget gene−CTLso recA)^].

To evaluate if *recA* could be used as a reference gene, Lso level in psyllids was quantified: a standard curve was prepared using a plasmid containing the Lso 16S rDNA target region at a known concentration (plasmid mass/L) using specific Lso 16S rDNA primers LsoF and HLBr (Levy et al., 2011; Nachappa et al., 2014). Five nanograms of DNA from each psyllid colonie were used to perform PCR reactions as described above. Correlation analyses between *recA* expression and Lso levels (16S rDNA CT levels) were performed.

### Data analysis

Insect development parameters were analyzed using SAS v9.2 (SAS Institute, Gary, NC) procedure PROC GLM to determine if there were significant difference among Lso-free, LsoA, and LsoB infected insects. Egg viability and nymphal survival were arcsine transformed prior to analysis. The significant differences in nymphal survivorship, and female oviposition among different Lso haplotype-infected insects were defined using LSD test with *p* ≤ 0.05. ANOVA tests were performed to evaluate differences of gene expression between Lso A and Lso B haplotypes in female and male insects (four treatments). Pairwise *t*-tests were performed when an overall significance was found.

## Results

### Lso haplotype effect on vector fitness

**Figure 1:**
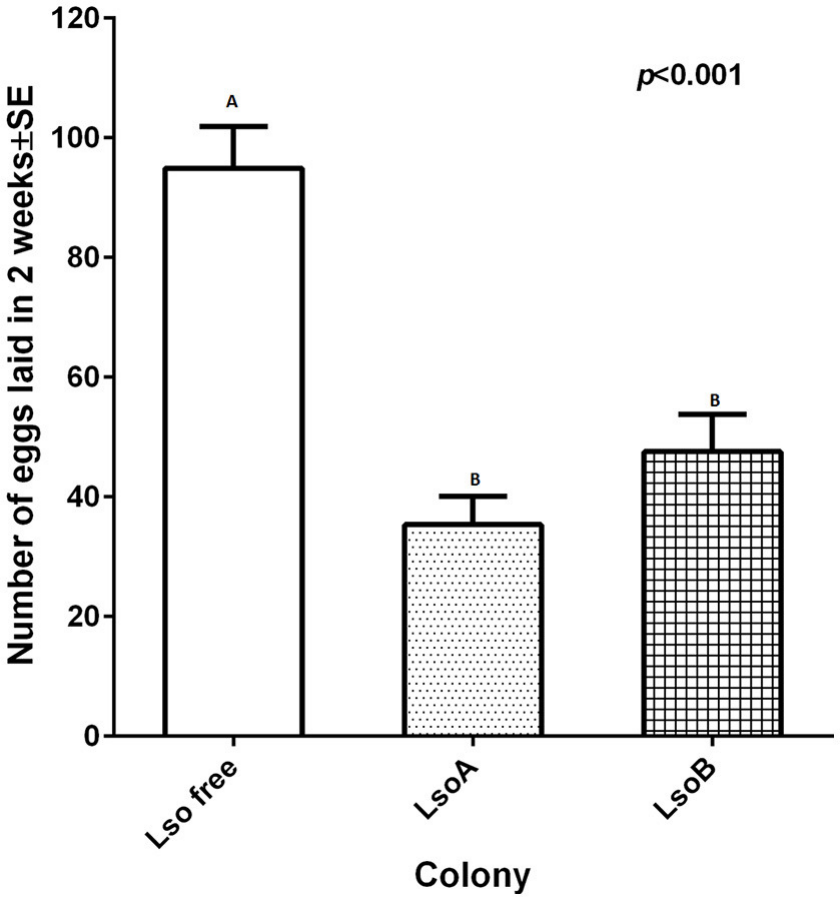
**Two-week oviposition by individual females from each colony**. One couple of insects was allowed to mate and to lay eggs for 2 weeks. Each column represents the mean and standard error of the number of eggs laid by individual females. Different letters indicate statistical differences between insect classes at *p* < 0.05 using LSD *t*-test.

To investigate the influence of Lso haplotype on oviposition, newly emerged adults of the LsoA, LsoB and Lso-free colonies were confined to a single leaf on tomato plants for 2 weeks. On average, 95 ± 7 eggs per Lso-free female were laid in 2 weeks; however, there were less than 50 eggs laid per Lso-infected female (Figure 1). Lso A- and Lso B-infected females had significantly lower oviposition than Lso-free females [*F*_(2, 33)_ = 25.95, *p* < 0.001]. However, no significant difference in oviposition was found between the two Lso-infected colonies (*p* = 0.8889).

**Figure 2:**
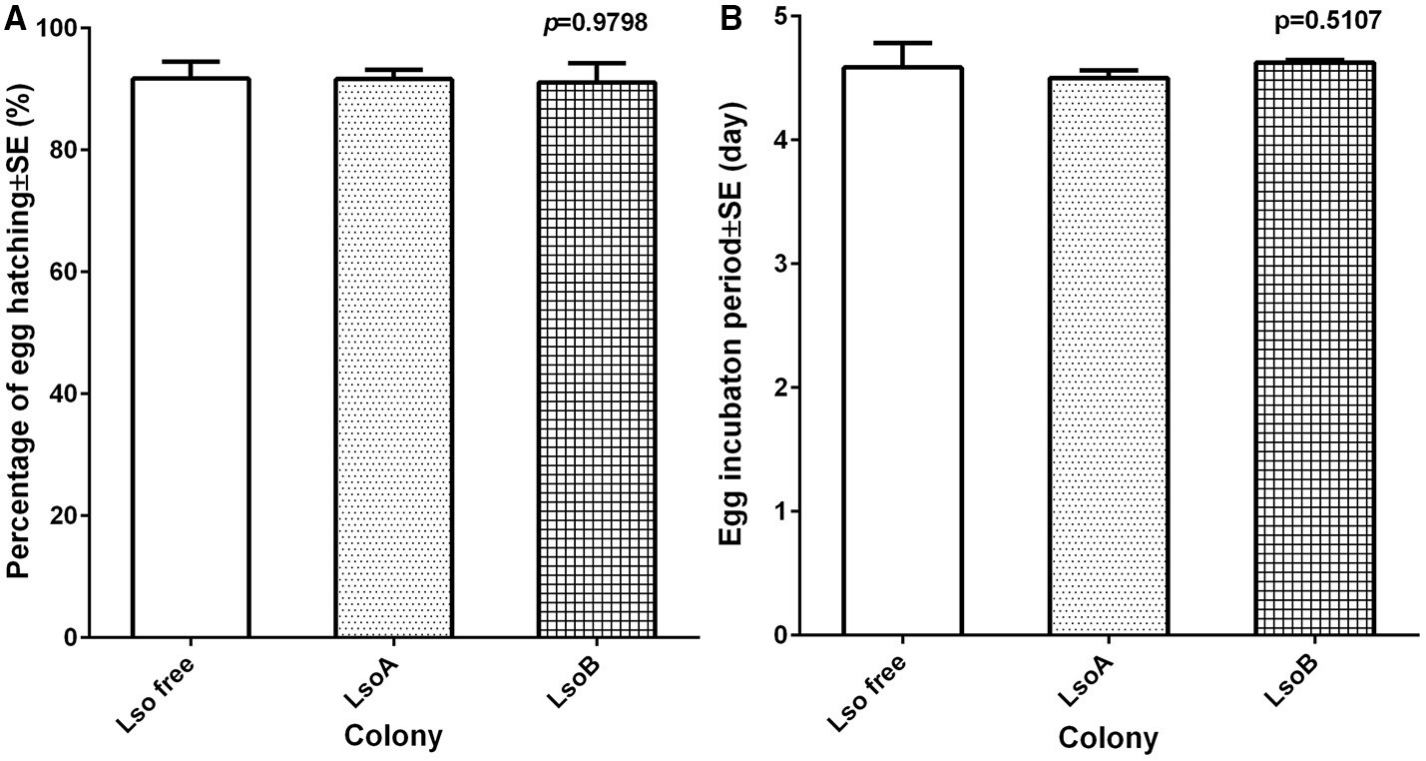
**Egg viability and average incubation time of each psyllid colony under different haplotype infection**. **(A)** The percentage of egg hatch was defined as the number of nymphs hatched out of the total number of eggs deposited. Each bar represents the mean and standard error of the percentage of egg hatching of *n* = 6 experimental replicates. **(B)** The egg incubation time was referred to the time period between the first oviposition and the appearance of first nymph. Each bar represents the mean and standard error of the egg incubation period in days of *n* = 6 experimental replicates.

Egg viability was determined as the percentage of eggs hatching (number of nymphs hatched out of the total number of eggs deposited). No significant differences were observed in egg viability among the Lso-free, LsoA, and LsoB potato psyllid colonies [*F*_(2, 15)_ = 0.02, *p* = 0.9825] (Figure 2A). On average, more than 90% of the eggs hatched independently of Lso presence or Lso haplotype based on three biological replicates for each treatment.

Egg incubation time was defined as the time elapsed between first oviposition and the appearance of the first nymph. No significant differences in egg incubation time were found [*F*_(2, 15)_ = 1.52, *p* = 0.2501] among the three colonies. Average egg incubation time was 4.57 days (Figure 2B).

**Figure 3:**
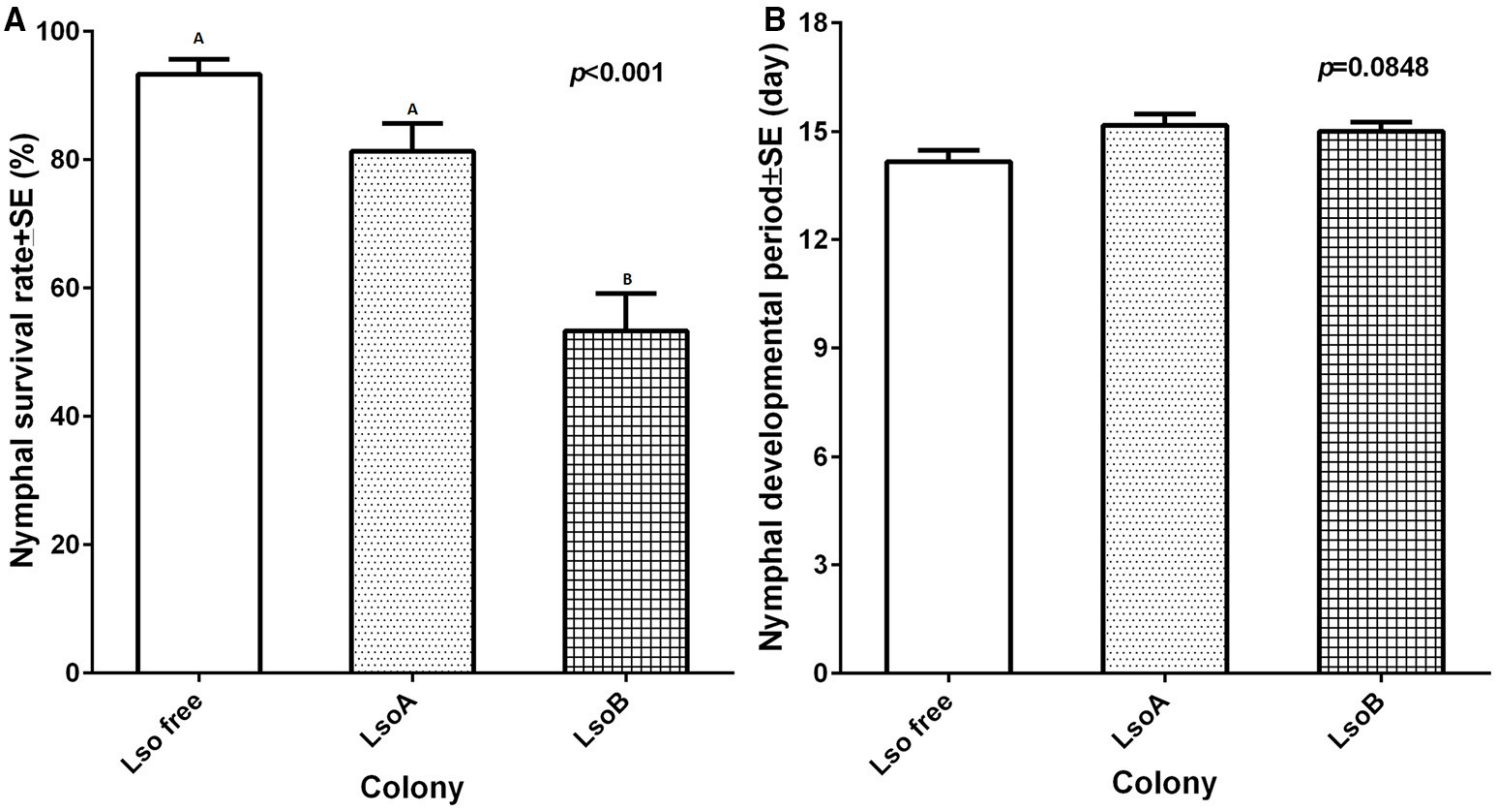
**Nymphal survival percentage and average immature developmental time of each colony. (A)** The percentage of nymphal survival was calculated as the number of emerged adults out of the total number of hatched eggs. Each bar represents the mean and standard error of nymphal survival percentage of *n* = 6 experimental replicates. Letters indicate statistical differences between insect classes at *p* ≤ 0.05 using LSD *t*-test. **(B)** Immature potato psyllid development time was measured as the time between the appearance of first instar and first adult emergence. Each bar represents the mean and standard error of the immature development period in days of *n* = 6 experimental replicates [*F*_(2, 15)_ = 3.37, *p* = 0.0619].

Potato psyllid nymphal survival percentages were significantly different among different colonies [*F*_(2, 15)_ = 21.90, *p* < 0.001]. The lowest percentage of nymphal survival was measured in the LsoB colony (average 53.35 ± 5.82%) compared to the LsoA and Lso-free colonies (81.37 ± 4.31 and 93.36 ± 2.31%, respectively; Figure 3A).

Development time of immature potato psyllid was measured as the time elapsed between the appearance of the first nymph and first adult emergence. Although Lso-free potato psyllid colony had the shortest measured immature development time (14.17 ± 0.31 days) compared with the Lso-infected colonies, LsoA (15.17 ± 0.30 days), and LsoB (15.00 ± 0.20 days; Figure 3B), there was no significant difference among the three colonies [*F*_(2, 15)_ = 3.37, *p* = 0.0619].

### Transcriptome analysis

To investigate Lso gene expression when associated with its vector, transcriptome sequencing of newly emerged females and males from the LsoA and LsoB colonies was performed. Over 70 million reads were obtained from each sample (Table 2).

**Table 2:** **Summary statistics of the global sequencing and read mapping performed as in Ibanez et al. (2014)**.

	**LsoA females**	**LsoA males**	**LsoB females**	**LsoB males**
Total reads	73,646,461	88,344,688	86,818,944	92,142,978
Mapped to bacterial genomes	369,784 (0.5%)	412,625 (0.47%)	980,283 (1.13%)	432,106 (0.47%)
Mapped to Lso genomes	110,061	111,607	467,779	159,120

**Figure 4:**
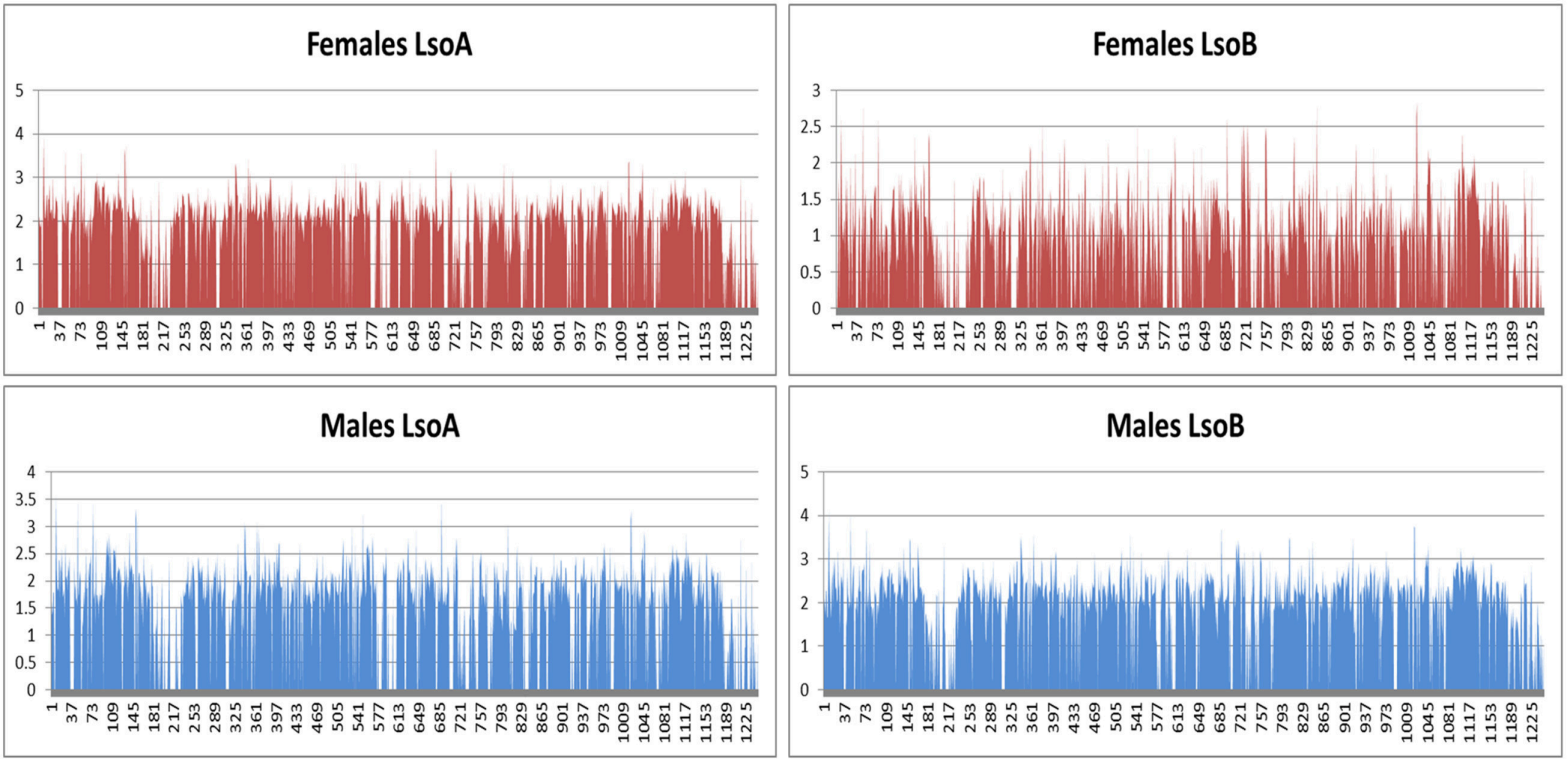
**Gene expression across Lso genome**. The *x*-axis shows the gene order in Lso B genome and the *y*-axis shows log(RPKM+1) value for each gene. Values for rRNA were changed to 0 for readability purposes.

Except for the females harboring Lso B sequencing, 0.5% of the reads mapped to the bacterial genomes (Nachappa et al., 2011). Higher percentage of reads mapped to the bacterial genomes in the females harboring Lso B sample. Analyses of the mapped reads revealed a high number of reads mapping to bacterial rDNA, therefore, in this sample bacterial rRNA depletion failed which in turn affected gene expression results for this sample. In spite of this rRNA depletion failure, similar patterns of gene expression across the Lso genome (Lin et al., 2011) were found for all samples (Figure 4).

The most expressed gene in each library was CKC_RS05865, a bacterial RNase P involved in maturation of the 5′ end of transfer RNA (Kazantsev and Pace, 2006). In all transcriptomes, heat shock and chaperones proteins were among the 10 most expressed coding sequences (CDS) (Table 3). CKC_RS02590, encoding flagellin, and CKC_RS00710, and CKC_RS00705, encoding pilus assembly proteins, were highly expressed in all libraries while CKC_RS00720, another pilus assembly protein was highly expressed in Lso A libraries, but expressed at lower levels in Lso B libraries.

**Table 3:** **Top 10 most expressed genes in each library after mapping to Lso B genome**.

	**Rank in female LsoA**	**Rank in male LsoA**	**Rank in female LsoB**	**Rank in male LsoB**
CKC_RS05865 Bacterial RNase P	1	1	1	1
CKC_RS00050 Hypothetical protein	2	3	7	2
CKC_RS03215 Hypothetical protein	3	2	6	6
CKC_RS00720 Pilus assembly protein	4	6	942	158
CKC_RS00350 Heat-shock protein	5	5	8	4
CKC_RS00710 Pilus assembly protein	6	7	164	16
CKC_RS00220 Hypothetical protein	7	4	4	3
CKC_RS00705 Pilus assembly protein	8	10	117	12
CKC_RS01705 Membrane protein	9	14	12	10
CKC_RS01605 Membrane protein	10	11	24	8
CKC_RS04735 Molecular chaperone GroES	11	8	2	7
CKC_RS02590 Flagellin	13	9	32	38
CKC_RS03915 Hypothetical protein	268	80	3	17
CKC_RS04730 GroEL	12	13	5	5
CKC_RS03350 Hypothetical protein	UN	UN	9	14
CKC_RS03385 Hypothetical protein	875	UN	10	57
CKC_RS02510 Hypothetical protein	15	16	13	9

*All genes without any mapped reads were ranked UN*.

Several genes annotated as hypothetical proteins were among the most expressed CDS in all libraries (CKC_RS00050, CKC_RS03215, CKC_RS00220, and CKC_RS02510); while CKC_RS03350 and CKC_RS03385, two genes encoding hypothetical proteins and sharing 91% of identity with each other, were highly expressed in Lso B libraries, but not in Lso A libraries or expressed at lower levels (Table 3). A CDS with 92% similarity to CKC_RS03350 is present in Lso A genome (AP064_03210).

To compare Lso A and Lso B transcriptomes, genes were ranked based to their expression level using their RPKM value. Several genes showing differences in expression between Lso A and Lso B were identified, and 28 genes were selected because they were expressed at relatively high levels in Lso B, but expressed at lower levels (or not expressed) in Lso A (Table 4). Several of those genes (22) had neighbors with similar expression patterns.

**Table 4:** **List of 28 genes showing higher expression in LsoB than LsoA insects**.

**Gene ID and annotation**	**Rank in LsoA females**	**Rank in LsoA males**	**Rank in LsoB females**	**Rank in LsoB males**	**Most similar gene in Lso A genome (% identity)**
CKC_RS00175 Hypothetical protein	UN	UN	29	48	AP064_02880 (78%)
CKC_RS02785 Hypothetical protein	UN	UN	17	31	NONE
CKC_RS02790 Hypothetical protein	UN	UN	52	94	NONE
CKC_RS03175 Hypothetical protein	UN	UN	263	771	NONE
CKC_RS03350 Hypothetical protein	UN	UN	9	14	AP064_03210 (92%)
CKC_RS03355 Hypothetical protein	961	914	26	25	AP064_03205 (81%)
CKC_RS03360 hypothetical protein	UN	UN	77	66	AP064_03200 (69%)
CKC_RS03385 Hypothetical protein	875	UN	10	57	AP064_03210 (69%)
CKC_RS03390 Hypothetical protein	971	933	28	160	AP064_03205 (82%)
CKC_RS03400 Hypothetical protein	987	944	321	627	AP064_03195 (86%)
CKC_RS03405 Hypothetical protein	271	818	46	29	NONE
CKC_RS03770 Hypothetical protein	730	615	111	347	AP064_03790 (96%)
CKC_RS03775 Head protein	932	955	549	541	AP064_03795 (99%)
CKC_RS03780 Hypothetical protein	993	881	249	552	AP064_03800 (98%)
CKC_RS03785 Phage portal protein	959	922	276	526	AP064_03805 (99%)
CKC_RS03790 DNA packaging protein	939	937	307	615	AP064_03810 (99%)
CKC_RS03885 Hypothetical protein	UN	UN	181	425	AP064_03555 (72%)
CKC_RS03890 Hypothetical protein	943	UN	41	30	AP064_03555 (86%)
CKC_RS04235 Hypothetical protein	UN	UN	25	13	NONE
CKC_RS04365 Hypothetical protein	UN	UN	31	73	AP064_04420 (73%)
CKC_RS04370 Hypothetical protein	UN	912	58	148	AP064_04425 (64%)
CKC_RS05155 50S ribosomal protein L29	689	879	73	238	AP064_04705 (96%)
CKC_RS05350 Hypothetical protein	812	938	491	259	AP064_01410 (70%)
CKC_RS05630 Hypothetical protein	966	710	65	183	AP064_05100 (91%)
CKC_RS05635 Hypothetical protein	UN	UN	398	470	NONE
CKC_RS05640 Hypothetical protein	985	UN	189	379	AP064_05100 (73%)
CKC_RS05645 Hypothetical protein	UN	UN	571	354	AP064_05105 (83%)
CKC_RS05825 Hypothetical protein	UN	UN	467	204	AP064_05115 (55%)

*All genes without any mapped reads were ranked UN*.

Five genes (CKC_RS02785, CKC_RS02790, CKC_RS03175, CKC_RS04235, and CKC_RS05635) were expresses in Lso B but not in Lso A libraries, and could not be found in the Lso A genomes. However, for other genes such as CKC_RS03350, CKC_RS03360, CKC_RS03885, CKC_RS04365, CKC_RS05645, and CKC_RS05825, genes with a high degree of similarity were identified in the Lso A genome. While the functions of those genes remain unknown, they are interesting candidates potentially associated with the measured differences in psyllid fitness between the colonies harboring each Lso haplotype.

CKC_RS03405, encoding a hypothetical protein in Lso B genome, had no homolog predicted in Lso A genome. Interestingly, reads from the Lso B and Lso A libraries mapped to this gene (Table 4). To locate a similar unpredicted ORF in Lso A genome, we used the sequence of the encompassing neighboring genes, CKC_RS03400 and CKC_RS03410, which were similar to Lso 7A AP064_03190 and AP064_03195 genes. Using BlastX analysis, a non-predicted putative ORF encoding an 81 amino acid long protein with 67% similarity to CKC_RS03405 was found in this region.

Other genes showing differences in expression between Lso A and Lso B were associated with the phage-related regions (Table 4). Those regions had been identified previously as differing between Lso haplotypes and potentially playing a role in pathogenicity (Thompson et al., 2015).

Interestingly, a few genes appeared as being expressed at higher levels in Lso A than Lso B, in spite of using the Lso B genome as reference. Some of those genes were CKC_RS00840, CKC_RS00145, CKC_RS02020, CKC_RS00890, or CKC_RS05650, all of them encoding hypothetical proteins.

To validate bioinformatic analyses, RT-qPCRs for 4 Lso genes were performed using each of the total RNAs that were used for rRNA depletion and pooling for sequencing. A linear correlation analysis of the relative expression levels between each RT-qPCR data for each experiment and the RPKM values was implemented. A strong Pearson correlation coefficient *r* = 0.81 was found between the bioinformatics results and the different biological replicates.

### Effector prediction analysis

The S4TE software predicted 23 type IV effector genes by using score 5 as threshold as shown in Table 5. Two genes were predicted with highest score at 8, CKC_RS05675 and CKC_RS00980, they encode hypothetical proteins. CKC_RS04080, CKC_RS03550, CKC_RS05565, and CKC_RS01780 (4 out of 23 genes) were predicted with score 7. CKC_RS04080 is a chemotaxis sensory transducer protein while CKC_RS03550, CKC_RS05565, and CKC_RS01780 are annotated as hypothetical proteins. There were 9 and 8 genes predicted with scores 6 and 5, respectively (Table 5). Among those genes, 6 of them are hypothetical proteins, 3 genes are involved in purine and amino acid synthesis (CKC_RS01950, CKC_RS05235, and CKC_RS00820), and 3 genes are involved in DNA repair and DNA restriction and modification (CKC_RS00225, CKC_RS01855, and CKC_RS00790).

**Table 5:** **List of predicted effector genes identify by S4TE (threshold score > 5)**.

**Gene**	**Protein description**	**S4TE score**
CKC_RS05675	Hypothetical protein	8
CKC_RS00980	Hypothetical protein	8
CKC_RS04080	Chemotaxis sensory transducer	7
CKC_RS03550	Hypothetical protein	7
CKC_RS05565	Hypothetical protein	7
CKC_RS01780	Hypothetical protein	7
CKC_RS04955	Hypothetical protein	6
CKC_RS03370	Hypothetical protein	6
CKC_RS01290	DNA translocase FtsK	6
CKC_RS05235	Leucyl/phenylalanyl-tRNA–protein transferase	6
CKC_RS03655	Hydroxymethylglutaryl-coenzyme A (HMG-CoA) reductase	6
CKC_RS02175	Peptidyl-prolyl cis-trans isomerase protein	6
CKC_RS01950	Bifunctional phosphoribosylaminoimidazolecarboxamide formyltransferase/IMP cyclohydrolase: purH	6
CKC_RS03915	Hypothetical protein	6
CKC_RS02450	Preprotein translocase subunit SecA	6
CKC_RS00880	Hypothetical protein	5
CKC_RS00225	Excinuclease ABC subunit C	5
CKC_RS00885	Hypothetical protein	5
CKC_RS05560	Hypothetical protein	5
CKC_RS01855	Type I restriction-modification system, M subunit	5
CKC_RS00790	Ribonuclease E	5
CKC_RS00820	Glycyl-tRNA synthetase subunit alpha: glyQ	5
CKC_RS00375	30S ribosomal protein S20	5

### Evaluation of S4TE-predicted effector gene expression by qPCR

To evaluate the S4TE-predicted effectors gene expression of Lso A and Lso B associated with psyllids, primers for the putative effectors were designed using Lso B genome. Primers were tested on DNA from LsoA and LsoB colonies. All primers amplified Lso A and Lso B DNA except for CKC_RS04955 which only amplify in Lso B. Based on dissociation analyses, seven primer pairs were excluded from further use (CKC_RS00980, CKC_RS02450, CKC_RS05560, CKC_RS01855, CKC_RS00790, CKC_RS00820, and CKC_RS00375).

To evaluate if *recA* could be used as a reference gene for RT-qPCR analyses, Lso density and *recA* expression were compared in different samples. For both sexes, a strong Pearson correlation coefficient r > 0.96 was found between *recA* expression levels and Lso density (Figure S1).

**Figure 5:**
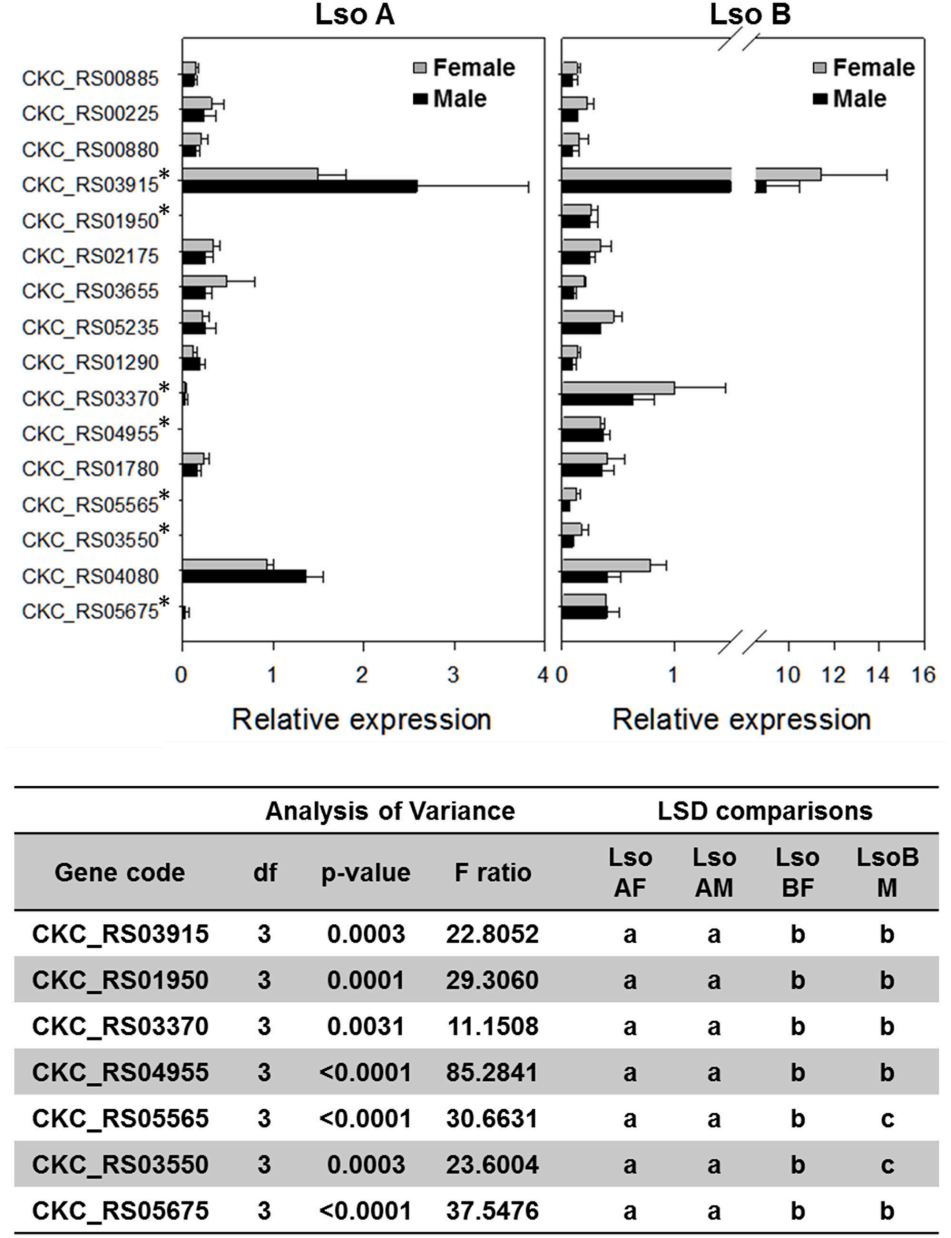
**Gene expression of putative type IV effectors in Lso A and Lso B haplotypes based on RT-qPCR**. *Show genes for which significant differences in gene expression between Lso A and Lso B were found following ANOVA tests and subsequent pairwise *t*-test *post-hoc* analyses.

Gene expression analyses of the 16 genes from the S4TE-predicted effectors gene list were performed from the total RNA of 1-day old Lso A-infected female and male, and Lso B-infected female and male psyllids (Figure 5). There were four genes (CKC_RS03550, CKC_RS05565, CKC_RS04955, and CKC_RS01950) for which expression in Lso A samples was undetectable. Similar expression pattern was found in the transcriptome for CKC_RS04955. Analysis of the Lso A genome after publication of the sequence (Zheng et al., 2014; Thompson et al., 2015), confirmed the absence of this gene in Lso haplotype A genome. CKC_RS01950 had similar relative expression levels in Lso A and Lso B transcriptomes. The differences between RT-qPCR and transcriptome data were linked to a SNP in the sequence of the reverse primer which did not match Lso A sequence. CKC_RS05565 and CKC_RS03550 encoded both hypothetical proteins. While based on RT-qPCR analyses these genes were expressed only in Lso B, similar expression levels in Lso A and in Lso B were found in the transcriptome. To validate the bioinformatic analyses, RT-qPCR for CKC_RS03550 with the RNAs used for Illumina library construction were performed and surprisingly yielded only expression in Lso B.

Three other genes (CKC_RS05675, CKC_RS03370, and CKC_RS03915) had higher expression in Lso B than in Lso A. Differences in relative expression ranged from at least 10 times higher in Lso B than Lso A for CKC_RS05675 (LsoB-Female = 0.40 ± 0.038, LsoB-Male = 0.39 ± 0.002; LsoA-Female = 0.03 ± 0.015, LsoA-Male = 0.003 ± 0.0003) and for CKC_RS03370 (LsoB-Female = 0.63 ± 0.066 and LsoB-Male = 1.00 ± 0.15; LsoA-Female = 0.033 ± 0.006 and LsoA-Male = 0.036 ± 0.0035); to 4 times higher in Lso B (LsoB-Female = 9.0 ± 0.49 and LsoB-Male = 11.43 ± 0.97) than Lso A (LsoA-Female = 2.59 ± 0.41 and LsoA-Male = 1.50 ± 0.10) for CKC_RS03915. Relative expression for these three genes was also higher in Lso B than Lso A in the transcriptome analysis. CKC_RS03915, encodes a hypothetical protein with a signal peptide ending at position 24-25, it was the gene with the highest expression level among the effector genes in the transcriptome.

No differences of expression between Lso A and Lso B were found for nine genes (CKC_RS04080, CKC_RS01780, CKC_RS01290, CKC_RS05235, CKC_RS03655, CKC_RS02175, CKC_RS00880, CKC_RS00225, and CKC_RS00885) based on RT-qPCR and transcriptomic analyses.

The expression level of CKC_RS04230 and CKC_RS02605 was also determined by RT-qPCR. They were selected among the 92 predicted putative effectors in the EffectiveDB database. They encoded proteins with signal peptides and were predicted to have an ER localization. CKC_RS04230 is a hypothetical protein, and its relative expression was higher in LsoB psyllids than in LsoA psyllids based on RT-qPCR (1.09 ± 0.24 and 0.83 ± 0.08 for LsoB females and males, and 0.04 ± 0.018 and 0.01 ± 0.0008 for LsoA females and males, respectively) and transcriptome analysis. No differences of expression between Lso A and Lso B were found for CKC_RS02605, annotated as chemotaxis protein.

Gene expression of the predicted effectors obtained by RT-qPCR and transcriptome were compared. A linear correlation analysis of the relative expression levels was performed and strong Pearson correlation coefficient *r* = 0.8077 was found between the bioinformatics results and the independent biological replicates.

## Discussion

*Liberibacter* bacteria are emerging as major threats to many crops world-wide (Haapalainen, 2014). Our understanding of their interactions with plants and their psyllid vectors remains limited, in particular due to their fastidious nature. Recently, the existence of five Lso haplotypes, two of which (Lso A and Lso B) are associated with solanaceous crops and potato psyllids in the Americas was published (Nelson et al., 2011, 2013; Alfaro-Fernández et al., 2012; Lin et al., 2012). This genetic diversity can be used to gain insight into the role of different genes and/or metabolic pathways on host adaptation. The objective of this paper was to assess the effect of each Lso haplotype on their insect vector and to identify differences in bacterial gene expression in association with the insect vector.

Previously, Lso A and Lso B double infected potato psyllids had been shown to have lower oviposition and nymphal survival than Lso-free psyllids on tomato plants (Nachappa et al., 2012b, 2014). In the current study, Lso-infected insects had lower oviposition than Lso-free females (Figure 1), but Lso infection had no effect on percentage of egg hatching, egg incubation time and nymphal development time (Figures 2A,B, 3B). Interestingly nymphal survivorship was severely affected by the presence of Lso B haplotype, while the survival of Lso A-infected nymphs was not significantly different than that of Lso-free nymphs (Figure 3A). Even though these results confirmed that Lso negatively affects potato psyllid population growth rate (Nachappa et al., 2012b, 2014), the differences of nymphal survivorship between Lso A- and Lso B-infected psyllids indicated that the presence of Lso B had a more dramatic effect on vector population growth than that of Lso A. Furthermore, these results imply that Lso B is more pathogenic to its vector than Lso A. The effects of Lso haplotypes on other psyllid populations might depend on the psyllid haplotype; this needs to be investigated.

In order to understand differences in gene expression between Lso A and Lso B that could be associated with differences of pathogenesis, a transcriptome analysis of Lso A and Lso B in association with the vector was conducted. To avoid potential changes in Lso gene expression in response to insect physiological changes related to insect age or sex, transcriptomic analyses were performed using 1-day-old female and male adults separately. Overall, similar gene expression profiles were obtained for each Lso haplotype (Figure 4). All libraries were characterized by high expression level of chaperone and heat-shock proteins, which are produced abundantly by insect endosymbionts including Lso (Ishikawa, 1984; Charles et al., 1997; Stoll et al., 2009; Ibanez et al., 2014).

Lso flagellin was highly expressed in all libraries, but interestingly it was expressed at higher levels in the Lso A than in the Lso B libraries. Lso flagellin has been shown to induce innate immune responses in *Nicotiana benthamiana* while induction in tobacco, tomato, and potato plants was weak (Hao et al., 2014). The effect of this protein on the vector has not been studied so far and it would be interesting to test whether this microbe-associated molecular pattern triggers immune responses in psyllids.

Several pilus assembly proteins were among the highly expressed genes, one of which, CKC_RS00720, was expressed at high levels in Lso A but expressed at lower levels in Lso B. Type IV pilus secretion system is involved in secretion of bacterial virulence factors that can have a role in host recognition and attachment, invasion, and biofilm formation (Rego et al., 2010). The existence and involvement of this system in *Liberibacter* pathogenicity has been suggested (Duan et al., 2009; Lin et al., 2011; Zhang et al., 2011; Kuykendall et al., 2012), and differences among Lso haplotypes in effector secretion systems might have an effect in their pathogenicity.

In order to assess if differences in type IV effectors between Lso A and Lso B could be associated with virulence and pathogenicity to its vector, S4TE software was used to predict type IV effectors. S4TE predicts and ranks type IV effectors candidates based on a combination of 13 distinctive features including homology to known effectors, homology to eukaryotic domains, presence of subcellular location signals or secretion signals (Meyer et al., 2013). A total of 23 putative effectors were found with a score over the threshold of Five. Since at that time no Lso A genome had been sequenced, RT-qPCRs were performed to test if there were differences in gene expression between Lso A and Lso B. Among the tested genes, 4 genes were only expressed in Lso B. Analysis of the published Lso A genomes (Zheng et al., 2014; Thompson et al., 2015) and the transcriptome data confirmed that one of them, CKC_ RS04955, is not expressed in Lso A. Three other putative effectors (CKC_RS05675, CKC_RS03370, and CKC_RS03915) had higher relative expression in Lso B than Lso A.

While Lso A and Lso B haplotypes share a high degree of similarity, this study has revealed differences in the expression of genes that could be implicated on host adaptation and pathogenicity. These differences of expression between Lso A and Lso B could arise from gain/loss of genes or be the consequence of genomic rearrangements resulting in changes of gene expression. Although RT-qPCRs can be used to compare gene expression of candidate genes between different haplotypes, a whole genome approach could be more informative because primer design can be challenging and lead to false positive/negative results without the correct genome sequence. For example, CKC_RS01950 was identified as a putative type IV effectors and RT-qPCRs revealed that this gene was not expressed in Lso A. CKC_RS01950 encoded for bifunctional phosphosylaminoimidazolecarboxamide formyltransferas/IMP cyclohydrolase which is involved in purine biosynthesis protein. Mutation of this gene in *Xanthomonas oryzae* pv. *oryzae* and in *Pectobacterium carotovorum* subsp. *carotovorum* resulted in virulence deficiency (Chatterjee and Sonti, 2005; Lee et al., 2013). Therefore, a difference in the expression level of this gene between Lso A and Lso B could be related to differences in pathogenicity. However, our transcriptomic analysis revealed that this gene is not differentially expressed.

A transcriptomic approach can help improve bacterial genome annotation (Ibanez et al., 2014) or gene prediction. Based on read mapping, genes that failed to be predicted *in silico* could be identified as the example presented here for CKC_RS03405.

It is interesting that none of tested S4TE-predicted effectors and other signal peptide containing proteins showed higher expression in Lso A than Lso B. The implication of this finding remains to be validated. Similarly, it would be important to evaluate if these genes are differentially expressed in the plant where they could be involved in plant infection.

## Author contributions

JY, PS, and FI conducted experiments, AM and OH helped with experiments, CN and DM performed S4TE predictions, JY, PS, JL, FI, DM, and CT analyzed and interpreted data, JL and CT conceived the study, all authors participated in the drafting and/or revision of the work.

### Conflict of interest statement

The authors declare that the research was conducted in the absence of any commercial or financial relationships that could be construed as a potential conflict of interest.

## Supplementary material

Figure S1 **Correlation between Lso density and *recA* expression level in psyllids harboring Lso A and Lso B**.
