# Supplementary figures and images for: Interactions “Candidatus Liberibacter solanacearum”—Bactericera cockerelli: Haplotype Effect on Vector Fitness and Gene Expression Analyses
Source: Front Cell Infect Microbiol. 2016 Jun 9;6:62. doi: 10.3389/fcimb.2016.00062 (PMC4899927; doi:10.3389/fcimb.2016.00062)

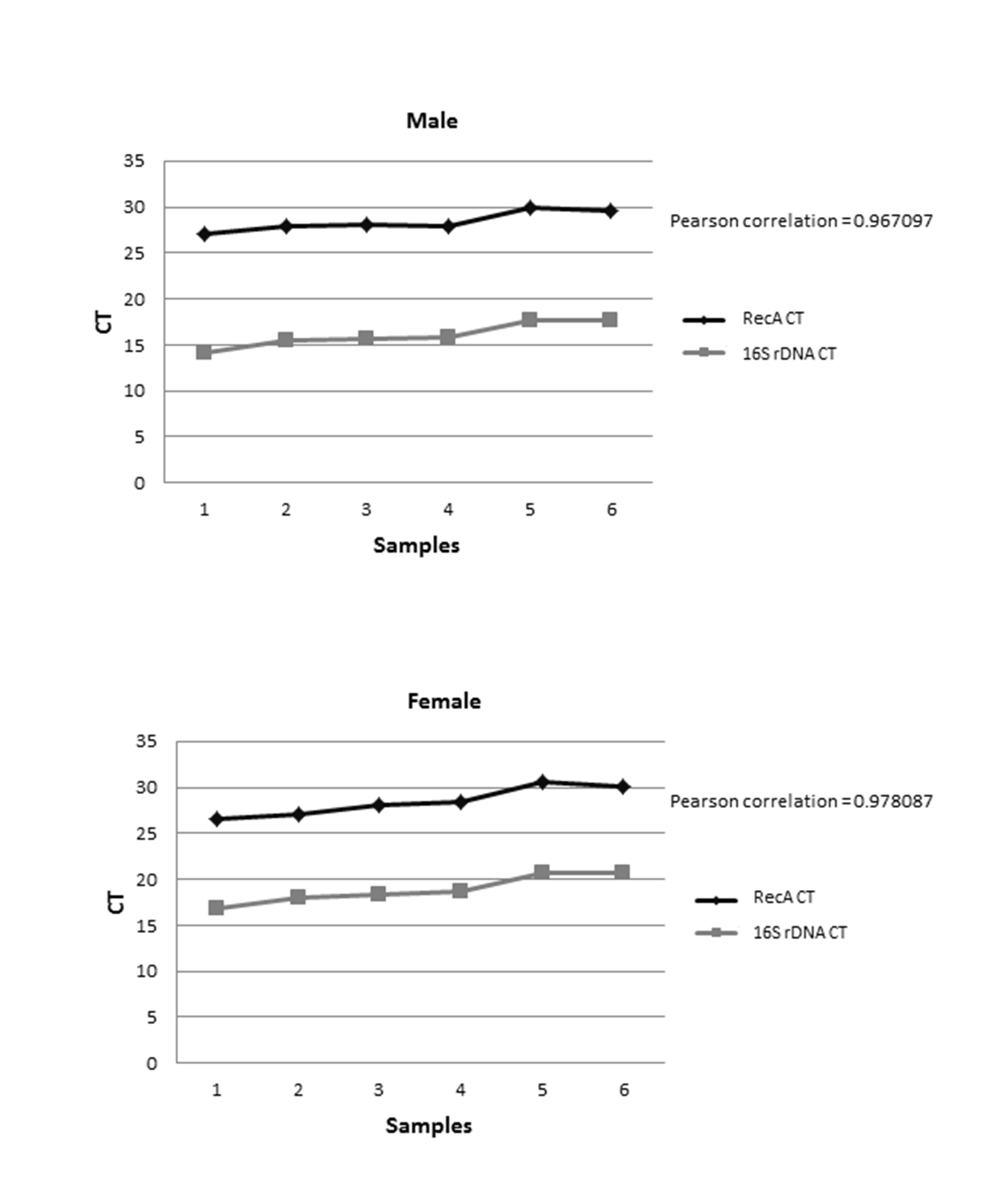

Supplement: Figure S1 — Correlation between Lso density and recA expression level in psyllids harboring Lso A and Lso B. [file Image1.tif]
